# Supplementary material for: Perceptions and behaviors of healthcare providers towards rehabilitation support to children with severe malaria-related disability in Ethiopia: A qualitative descriptive study using the Theoretical Domains Framework
Source: PLoS One. 2024 May 2;19(5):e0298769. doi: 10.1371/journal.pone.0298769 (PMC11065226; doi:10.1371/journal.pone.0298769)
Supplement: S1 Table — (DOCX) [file pone.0298769.s001.docx]

**S1 Table. Interview guide**

| **Interview Questions**  **Grand tour questions**   1. Could you tell me a little bit about yourself and your role in this health facility? 2. How would you describe your experience in caring children with malaria?   **General questions about severe malaria-related disability**   1. **Perceptions about the consequences of severe malaria** 2. How would you describe severe malaria? 3. What are your views and beliefs about the long-terms consequences of severe malaria? 4. **Understanding the nature of the behaviour** 5. Could you tell me about the various steps in how children with severe malaria are managed and supported in your health facility? 6. What does the management approach (protocol) of children with severe malaria look like in your health facility? 7. **Factors influencing the behavior of health care providers** 8. What opportunities are there to prevent severe malaria-related long-term impacts or to provide rehabilitation support for children with long-term consequences of severe malaria? 9. What do you think are the barriers and/ the challenges? |
| --- |

**Prompts questions grouped based on TDF domains.**

| **TDF domains** | **Prompt questions** |
| --- | --- |
| Knowledge | - Could you tell me about severe malaria-related components of disability? - Are you familiar with integrated approaches such as the International Classification of Functioning, Disability, and Health (ICF)? Please tell me about it. |
| Skills | - What skills are needed to address severe malaria-related disability? - Could you tell me about your skills (competences) related to the provision of rehabilitation support for children with severe malaria-related disability? |
| Social/Professional Role and Identity | - Do you think the prevention of severe malaria-related disability and provision of rehabilitation support are part of your professional role? Tell me more about these issues. |
| Beliefs about Capabilities | - Any difficulties or challenges in supporting children with severe malaria-related disability? |
| Optimism | - What are your ambitions in managing these problems? |
| Beliefs about Consequences | - What do you think are the benefits/advantages of using comprehensive approaches in addressing severe malaria-related disability? - What do you think are the disadvantages of using comprehensive approaches in addressing severe malaria-related disability? |
| Reinforcement | - Are there incentives? |
| Intentions | - Do you feel you have to do it? Why? |
| Goals | - What are your goals? |
| Memory, Attention and Decision Processes | - Is it something you do routinely? - Could you tell me about your decision process? |
| Environmental Context and Resources | - What institutional factors influence whether you provide rehabilitation support for children with severe malaria-related disability or not? - Are there tools available (e.g., specific clinical guidelines)? - Are there enough human resources? - Are there clear communication channels? - Are there enough physical resources? - Do you have enough time? - Does the working environment of the health facility have an effect? |
| Social influences | - Do you seek opinions of colleagues in preventing severe malaria-related disability and providing rehabilitation support? - What are the views of your colleagues? |
| Emotion | - Is the prevention of severe malaria-related disability and provision of rehabilitation support challenging to deal with? - Would you prefer to avoid this behavior? |
| Behavioral Regulation | - What would you need to prevent severe malaria-related disability and provide rehabilitation support? - Are there any protocols to facilitate the practice? |
