# Supplementary material for: Perceptions and behaviors of healthcare providers towards rehabilitation support to children with severe malaria-related disability in Ethiopia: A qualitative descriptive study using the Theoretical Domains Framework
Source: PLoS One. 2024 May 2;19(5):e0298769. doi: 10.1371/journal.pone.0298769 (PMC11065226; doi:10.1371/journal.pone.0298769)
Supplement: S2 Table — (DOCX) [file pone.0298769.s002.docx]

**S2 Table. Coding guide**

| **TDF Domains** | **Coding Guide** |
| --- | --- |
| **Knowledge** | - Health care providers’ existing knowledge about severe malaria-related disability, comprehensive approaches to identify the components of disability (e.g., understanding of severe malaria-related disability from the biopsychosocial perspective), prevention of severe malaria-related disability and provision of rehabilitation support |
| **Skills** | - The required skills or competences to prevent severe malaria-related disability and provide rehabilitation support |
| **Social/Professional role & identity** | - Health care providers’ description of expectations, professional roles, boundaries, responsibilities, job descriptions, etc., that include comparing of their roles and responsibilities with colleagues or other professionals in terms of preventing severe malaria-related disability and providing rehabilitation support. |
| **Beliefs about capabilities** | - Health care providers’ perceptions about their own competence or self-confidence on preventing severe malaria-related disability and providing rehabilitation support. - Perceptions about the difficulties or challenges in preventing severe malaria-related disability and providing rehabilitation support |
| **Optimism** | - The ambitions that are expressed in a way that they will successfully prevent severe malaria-related disability and provide rehabilitation support |
| **Beliefs about consequences** | - Health care providers’ perceptions about the advantages, disadvantages, and outcomes of preventing severe malaria-related disability and providing rehabilitation support |
| **Reinforcement** | - Financial or non-financial incentives that influence the healthcare providers’ behaviour on preventing severe malaria-related disability and providing rehabilitation support. - Other positive or negative mechanisms such as punishment or other disappointing circumstances |
| **Intentions** | - Any feeling or desire to properly prevent severe malaria-related disability and provide rehabilitation support, or to change the current behaviour that is related to the prevention of severe malaria-related disability and provision of rehabilitation support |
| **Goals** | - Any plans, commitments, priorities set by the healthcare providers to take actions that are related to the prevention of severe malaria-related disability and provision of rehabilitation support |
| **Memory, attention & decision processes** | - Health care providers’ decision process related to the prevention of severe malaria-related disability and provision of rehabilitation support that includes the factors that they consider (e.g., patient factors) for their decision making |
| **Environmental context and resources** | - Any institutional factor that influences the behaviour of health care providers on preventing severe malaria-related disability and providing rehabilitation support such as specific clinical guidelines, human resource, physical resource, time, working environment, etc. |
| **Social influences** | - Seeking opinions from other people about the prevention of severe malaria-related disability and provision of rehabilitation support or external influence from colleagues, other people and/or patients or the views of colleagues and other people |
| **Emotion** | - Any feelings related to the prevention of severe malaria-related disability and provision of rehabilitation support such as inconveniences, discomforts, unhappiness and/or the reverse aspect |
| **Behavioural regulation** | - Creating mechanisms or ways that help to prevent severe malaria-related disability and provide rehabilitation support such as short-term courses, trainings, updating oneself using online resources and/or related intentions. - Reported needs to prevent severe malaria-related disability and provide rehabilitation support (e.g., training, payment, other resources) - Individual level action plan and/or organizational guideline or specific clinical protocol that facilitate the prevention of severe malaria-related disability and provision of rehabilitation support |

**Coding rules**

1. Coding will be done according to the directed content analysis method using the published TDF domains.
2. Excerpts will be coded into the most relevant TDF domains based on their logical and conceptual connection.
3. If a single utterance relates to multiple TDF domains, coding will be done based on the main message of the expression.
4. In case of uncertainties, excerpts will be coded in multiple domains. In such cases, the excerpts will be marked with asterisks for further review and modifications.
5. A coding guide will be used to facilitate the coding process (see S2 Table).
