# Supplementary material for: Perceptions and behaviors of healthcare providers towards rehabilitation support to children with severe malaria-related disability in Ethiopia: A qualitative descriptive study using the Theoretical Domains Framework
Source: PLoS One. 2024 May 2;19(5):e0298769. doi: 10.1371/journal.pone.0298769 (PMC11065226; doi:10.1371/journal.pone.0298769)
Supplement: S3 Table — (DOCX) [file pone.0298769.s003.docx]

**S3 Table. The proportion of codes addressing prevention**

| **Domain/ theme (specific belief)** | **Example quote** | **Proportion of codes addressed**  **Prevention*** |
| --- | --- | --- |
| 1. **Knowledge** |  | **5.4** |
| "We do not know much about it" | "It is a little bit challenging to talk about this issue because it needs a follow-up or further investigation as we do not know much about it. To know such a case, children who have survived severe malaria should seek health care in our facility. ... but unfortunately, I cannot exactly tell what kind of health problems such a child might have developed" (R302).  "The long-term effects may require more research; I did not experience any health issues like these. I agree that malaria can have consequences, but I have not seen such cases. However, when you consider the scientific evidence about malaria's pathophysiology, there is no way it can be free of long-term complications" (R305).  "We do not know much about it, and I have not paid enough attention to it, to your surprise" (R306). | 5.4 |
| 1. **Skills** |  | **8.1** |
| "We need training to fill our gaps" | "We do not have specialized training to assess and manage these complications; we are managing some related problems using our clinical experiences, so we need additional training to fill our gaps" (R302).  "It would be fantastic to fill our skills gaps through training, emphasizing the types of consequences and management measures. I would be pleased to follow those approaches and deal with the problems" (R312). | **8.1** |
| 1. **Beliefs about consequences** |  | **56.2** |
| - 1. Beliefs about disease outcomes | "… if they arrive early, it is simple to cure: most recover quickly. Some children may suffer hypoglycemia or anemia, but both are treatable if caught early. On the other hand, the younger ones may die if they contract cerebral malaria. If they survive the death, I believe they need follow-up" (R301).  "It could harm their social skills, cause them to do poorly in school, and cause them to regress as a result. It's possible that their mental function might be impacted as well" (R304).  "Although we have not paid much attention to these issues, I believe the disease [severe malaria] has several long-term effects. Some suffer splenomegaly and anemia, and if they are frequently attacked, they become vulnerable to other diseases; some may also have developmental issues" (R313). | 20.4 |
| - 1. Beliefs about severity of illness | "I think we need to consider various factors while determining whether or not it is severe malaria. Most children cannot stand on their own and stumble when they attempt to do so. ... they are generally accompanied by a high-grade fever and low blood pressure. When sending a blood sample to a lab, the results usually show a high [malaria] parasite load. Most children develop anemia. ... they also lose concentration, as I already stated" (R305).  "Severity is not one thing; it has different features. For example, some cannot eat orally, and others suffer from seizures and loss of consciousness. In addition, the [malaria] parasite load is usually high in most children, and some become comatose" (R307). | 94.1 |
| 1. **Goals** |  | **54.3** |
| - 1. Considering such problems in the future | "If someone wants to work in this area in the future, I believe it is critical to think about the problem and strengthen actions related to it. I hope that every professional, up to the grassroots level, needs to understand how to deal with these issues and takes appropriate action" (R304).  "This issue is new, so it is better to give it due attention for the future. Although many people do not know much about the long-term impacts of severe malaria, we will work on all the required activities to identify cases. We will also try to manage the problems as much as we can" (R307). | 11.1 |
| - 1. Planning to work on preventive aspects | "My goal is to prevent children from being ill. So, I would like to focus on the preventative aspects. Why should people be made to suffer? Apart from the illness, there are medical costs, human resource issues, and ups and downs. So, I think it is better to focus on prevention" (R302). "As a health extension worker, I intend to focus on preventative measures" (R309). | 100 |
| 1. **Optimism** |  | **10** |
| I hope things will improve in the future | "I am hopeful that policies and strategies will be designed and implemented, with non-governmental organizations assisting in the process. Despite any shortcomings on the part of the implementers [health professionals], I am confident that things will be fine in the future" (R303).  "There is a window of opportunity for improvement. For example, there were no organ function tests in primary hospitals, but we do have that now. So, I hope things will improve in the future" (R305). "Things, in my opinion, will improve in the future. Researchers like you will present evidence to policymakers, who then might design policies and strategies to address the health problems associated with severe malaria's long-term consequences. For example, a guideline for dealing with these issues might be available" (R310). | 10 |
| 1. **Reinforcement** |  | **66.7** |
| - 1. Discouraging conditions | "You know what? When children develop complications, their parents usually take them to holy water and bring them to us when life-threatening conditions happen. So, my only option would be to refer them to a hospital, which made me feel desperate" (R306).  "Nothing motivates. I will do that because it is part of my responsibilities. When children do not improve, though, I am disappointed. So, it is not only a payment or incentive issue; it is more than that" (R311). "You are supposed to manage the complications [the community expects], but it is discouraging if you cannot" (R312). | 74.1 |
| - 1. Rewarding conditions | "... the first is internal motivation; the second is that performing your duties will make you happy. I mean, it feels pleasant on the inside. ... but above all, it inspires when the child regains his health" (R312). "Some organizations assist our activities, especially non-governmental ones. Sometimes the ministry of health also motivates professionals. For example, it gave a training chance for one professional last year" (R313). | 53.3 |
| 1. **Environmental context and resources** |  | **80.4** |
| - 1. Absence of relevant guideline | "No, there has not been a protocol for long-term issues until now. There is, however, one for treating acute malaria, about first-line medications, second-line medications, and so on" (R301).  "To be honest, the guideline we are using focuses solely on treating acute malaria. So, it is all about dealing with acute malaria at the health post, health center, and hospital levels" (R305).  "I have not seen a guideline for managing long-term consequences; I did not read one either. ... probably, there might be one, so I will look for it anyhow. ... but the fact is, I did not come across such a guideline as far as I remember" (R308). | 75 |
| - 1. Scarcity of required resources | "Pediatricians alone might not be enough. It could be helpful if there were clinicians who specialized in neurology. That is just one example from the perspective of human resources. On the other side, as some diagnostic devices such as CT-scan and MIR are absent, it is challenging to decide on the types and stages of the problems. So, generally, essential investigations and diagnostic modalities are incomplete" (R301).  "I think the long-term consequences need professionals specially trained for that purpose. However, unfortunately, we now lack such practitioners who can adequately assess and manage these issues" (R303).  "Our duty becomes more manageable when materials are complete, but this is not always the case. For example, we refer a mother with a febrile child when an RDT [Random Diagnostic Test for Malaria] is unavailable" (R309). | 76.2 |
| - 1. Systems and processes affecting treatment | "When new epidemics or pandemics emerge, malaria will become secondary or forgotten, and the resources will be transferred to the new challenges. Emerging health issues always divert the health sector's attention" (R301). "The referral system is weak. When we transfer patients to a hospital, we frequently experience a disconnect. The professionals there frequently do not see the referral paper and, as a result, do not provide us with feedback that prevents us from following up on the health conditions of the children who have survived" (R302). | 91 (91) |
| 1. **Social Influences** |  | **90.3** |
| - 1. Patients' beliefs | "When we refer some malaria survivors to higher-level health facilities for further investigation or better management, parents frequently refuse and insist that we do everything we can. ... but, of course, life in this district is difficult due to financial constraints" (R302). "I am worried about the children who have survived severe malaria, especially those who live in rural areas. ... unfortunately, regardless of their financial means, families' attention to children is often minimal, so this way of thinking may have a negative impact on the treatments and advice we provide" (R309). | 87 |
| - 1. Socio-cultural influences | "It is a deep-rooted belief! When they just brought the child, especially if he comes while conscious, they do not allow us to provide him injectable medications. They would disagree with parenteral routes unless they first visited the holy water treatment" (R306).  "Even if a child is shivering with an acute malarial attack, there is a strong desire to turn to traditional healers, holy water, or witches. When the child's condition worsens, it is common to blame "Satan" or an "evil spirit" for the child's problems. As a result, parents come to us when things get out of hand" (R313). | 92.3 |
| 1. **Social or professional role and identity** |  | **73.8** |
| - 1. Clinical practice inconsistencies among professionals | "Most children with severe malaria had been treated with oral anti-malaria drugs before arriving at our health facility, although they were meant to be referred immediately. As a result, some arrived after they had become comatose, and we usually had no choice but to refer them to the next higher level of care" (R203). "The problem, in my opinion, primarily begins in peripheral health facilities. I think health extension workers are not doing their jobs properly. For example, most problems may have been avoided or managed earlier if they raised community awareness and promptly referred severe cases" (R313). | 91.8 |
| - 1. Beliefs about scope of practice | "I believe it has something to do with my job. So, if I come into a problem like this, I will talk to colleagues or malaria experts at district health offices about supporting the children who are impacted" (R304). "When an activity is included in one of the 17 health extension packages, it is definitely within my scope of practice. So, for example, I can do preventive activities and treat mild cases, as these are within the packages. However, I am not usually the one that handles managing disabilities related to severe malaria" (R312). | 11.1 |
| - 1. We work together and the team spirit is good | "Physicians, nurses, and other health professionals are present as this is a hospital. I work with them, and we have a great team spirit. Nurses carry out whatever is expected of them in the absence of physicians, which is also true in the absence of nurses. ... we constantly collaborate and function as a team" (R305). "We collaborate and, for the most part, have a common understanding. For example, if a physician prescribes the incorrect dose, a pharmacy expert discusses it with the prescribing physician and makes the necessary corrections. Other professionals, such as nurses and laboratory technicians, also communicate and collaborate in the same way" (R308). | 88.2 |

*The proportion of data (codes) in a domain or sub-theme addressing the prevention of severe malaria-related disability (percentage was calculated as follows: number of codes addressing prevention of severe malaria-related disability within a domain or sub-theme divided by total codes within a domain or sub-theme multiplied by one hundred)
