# Supplementary material for: Perceptions and behaviors of healthcare providers towards rehabilitation support to children with severe malaria-related disability in Ethiopia: A qualitative descriptive study using the Theoretical Domains Framework
Source: PLoS One. 2024 May 2;19(5):e0298769. doi: 10.1371/journal.pone.0298769 (PMC11065226; doi:10.1371/journal.pone.0298769)
Supplement: Attachment — Submitted filename: Review draft.docx [file pone.0298769.s005.docx]

Manuscript is well written. This article is an attempt to delve into one of the major problems related to perception of health care givers in children having disabilities post severe malaria.

However, the sampling frame is not uniform and sampling units have wide variations. Overall sampling size is too small (only 13). It is a mixture of 3 healthcare facilities i.e District Hospital (3), specialised hospitals (3), primary healthcare (7). The three different health care facilities having different types of perception to handle these problems. It is one of the major limitations.

As there is a wide variation in sampling units, it is but natural that the perception in domain subtheme like knowledge, skill, beliefs and consequences, goals, reinforcement will so wide variation which is not acceptable.

Under these circumstances, there is every possibility that the inferences drawn and conclusions made may be having bias.

It is suggested that sample size may be increased so that each category of respondents are adequately represented. Possibility of subgroup analysis may also be attempted.
