# Supplementary material for: Perceptions and behaviors of healthcare providers towards rehabilitation support to children with severe malaria-related disability in Ethiopia: A qualitative descriptive study using the Theoretical Domains Framework
Source: PLoS One. 2024 May 2;19(5):e0298769. doi: 10.1371/journal.pone.0298769 (PMC11065226; doi:10.1371/journal.pone.0298769)
Supplement: Attachment — Submitted filename: Response to Reviewers.docx [file pone.0298769.s006.docx]

**Response to Reviewer 1**

| Reviewer’s comment/question | Authors’ response |
| --- | --- |
| As the sampling frame and sampling units have wide variation as well as sample size is too small. Under these circumstances, there is every possibility that the inferences drawn, and conclusions made may be having bias. It is suggested that sample size may be increased so that each category of respondents are adequately represented. Possibility of subgroup analysis may also be attempted. | Thank you so much for your valuable comments, and we understand your concerns. In contrast to research that applies theoretical data saturation, such as the grounded theory, this qualitative descriptive study did not attempt to fully describe all aspects regarding the perceptions and behaviors of healthcare providers, nor does the study aim to identify either consistency or substantial differences in the perceptions and behaviors of the various professionals included in the study, which could lead to a sub-group analysis. Instead, we focused on a comprehensive understanding of the phenomenon and identifying communalities. Including various professionals helped us enriched the data from different perspectives and contexts.  Therefore, participant recruitment was stopped after collecting a rich and varied account of the phenomenon under investigation by applying the concept of "information power" and the techniques recommended by Francis and colleagues (2010) for theory-informed qualitative studies, cited in the manuscript. No new information was obtained when three participants were interviewed following a preliminary analysis of the data obtained from the first 10 participants; therefore, continuing the data collection after the 13th interview was unnecessary. |

**Responses to Reviewer 2**

| Reviewer’s comment/question | Authors’ response |
| --- | --- |
| 1. Provide a clear/standard definition for severe malaria related disability in the context of this study -What is the scope? As mentioned on page 3 line 60 to 63 most published literature on malaria related complications are discussed in acute context. Is this disability limited to neuro-cognitive disability outside of acute complication of malaria? On page 3 lines 65 and 66 mentions known rehabilitation and interventions have focused on neurocognitive impairment. | Thank you so much for your valuable feedback.  Severe malaria is a laboratory-confirmed malaria characterized by one or more clinical symptoms or laboratory findings, including impaired consciousness, respiratory distress, multiple convulsions, prostration, abnormal bleeding, jaundice, hypoglycemia, acidosis, hyperlactatemia, renal impairment, hyperparasitemia, cerebral malaria, and severe malarial anemia. In line with the World Health Organization's standard definition of disability, any form of impairment (e.g., attention deficits), activity limitation (e.g., behavioral problems), and participation restriction (e.g., negative peer attitudes) that occurred because of this health condition was considered as severe malaria-related disability. Therefore, neurocognitive impairments are included but are not the only forms of severe malaria-related disability.  We included these standard definitions in the revised manuscript (page 3 line 50-56) in track changes. |
| 2. Was there prior consideration of other frameworks such as CFIR (widely used in low- and middle-income countries) before the selection of TDF in this context where there is no clear evidence based guideline/protocol for management of severe malaria related disability? Was any consideration that TDF and CFIR approach include determinants at both individual and collective level? Would combining the CFIR and TDF help to more fully define the multi-level nature of behavior change in healthcare organizations be a better approach than using either of them alone? | We compared other determinant frameworks, such as the active implementation framework, CIFIR, and PARIHS, before deciding on the TDF. We preferred TDF to other frameworks because our primary goal was to identify key theoretical constructs pertinent to healthcare providers' perceptions and behaviors towards rehabilitation support to children with severe malaria-related disability so that these constructs will be accessible for future large-scale quantitative studies and help develop strategies for effective implementation.  We agree that CFIR is a widely used framework in low- and middle-income countries and is among the top five most accessed articles in implementation science. However, we believe that, for the objective we formulated, our selection of the TDF was also appropriate, given that the TDF contains 33 behavior change theories that are accessible in 14 theoretical domains, which can capture healthcare providers' perceptions, behaviors, and (unlike most other behavioral theories) environmental and institutional factors.  We also agree that combining CIFR and TDF could help us more fully understand multi-level behavior change than using individual frameworks. However, we did not aim to fully describe all aspects of behavior change in our qualitative descriptive study. Instead, we focused on a comprehensive understanding of the phenomenon. We will consider combining the two frameworks in our future large-scale studies. |
| 3. For sampling-how was the number of 13 health workers arrived at? Representation of different cadres mentioned; did any health workers decline to participate and if so why? | Participant recruitment was stopped after collecting a rich and varied account of the phenomenon under investigation by applying the concept of "information power" and the techniques recommended by Francis and colleagues (2010) for theory-informed qualitative studies, cited in the manuscript. No new information was obtained when three participants were interviewed following a preliminary analysis of the data obtained from the first 10 participants; therefore, continuing the data collection after the 13th interview was unnecessary.  We used a purposive sampling technique based on criteria, and no participant declined recruitment or withdrew from participation after recruitment. |
| 4. In the section on “Trustworthiness” Comment on positionality of EE having established relationship with the participants over time and his involvement in analysis-would this introduce any bias and how was this minimized? | We used various methods to ensure trustworthiness, including prolonged engagement, audit trail, debriefing, member check, and reflexivity. Establishing relationships was part of the prolonged engagement and was necessary. However, we continuously reflected on the process and our feelings to minimize bias. As an interviewer, I paid particular attention to this aspect and documented a reflexivity statement during the data collection period. The reflexivity continued throughout the research process, including data analysis. To that end, the prolonged engagement contributed to obtaining rich data. It did not introduce significant bias to the extent that affected the findings, as we were aware of our actions and feelings through continuous reflexive journaling. However, we are not arguing that we avoided bias 100%, as complete bracketing is impossible.  We described this in the manuscript on page 10, lines 193-195, by adding a statement in track changes. |
| 5. In the analysis approach after codebook development and pilot by EE and colleague-was this followed by primary coding and secondary coding by different researchers? What was the approach to the resolution of queries or disagreement in coding-Were all researchers involved or some of them? | Primary and secondary coding was done by EE and NF using a coding guide, and the reliability of coding was checked first by proportion and then by Kappa statistics. The other team members were also involved in a discussion to resolve a few codes that the two coders disagreed on, which helped finalize the coding process.  We provided this description in the manuscript, page 8, lines 176-179 in track changes. |
| 6. Was any consideration given to health workers caring for children with preexisting conditions who may suffer more severe disability from malaria for example children with anemia due to different causes example sickle cell disease –prevalent in some East African countries like Kenya Uganda and Tanzania…Is this prevalent in Ethiopia? | In our study, severe malaria was defined as laboratory-confirmed malaria characterized by one or more clinical symptoms or laboratory findings, including impaired consciousness, respiratory distress, multiple convulsions, prostration, abnormal bleeding, jaundice, hypoglycemia, acidosis, hyperlactatemia, renal impairment, hyperparasitemia, cerebral malaria, and severe malarial anemia. Therefore, disability resulting from other con-existing conditions was not considered.  We did not study the prevalence of severe malaria-related disability; however, as malaria is prevalent in nearly 60% of the country (Ethiopia), a substantial number of children with severe malaria-related disability is expected. Our recently published qualitative study also gives clues for that ([Severe malaria-related disability in Ethiopian children from the perspectives of caregivers: an interpretive description study - PubMed (nih.gov)](https://pubmed.ncbi.nlm.nih.gov/37303154/)). |

**Responses to additional editor comments**

| **Editor comment** | **Authors’ response** |
| --- | --- |
| The authors need to increase the sample size of participants in the study as suggested by the reviewers. Also, various categories of health workers should be recruited to participate in the study. Also, STANDARD TOOLS AND RESEARCH METHODS SHOULD BE APPLIED IN THE STUDY. | Dear the editor, thank you so much for these additional comments.  Regarding the sample size, we believe that we had adequate sample size, given the purpose of our study and the methods we applied including the concept of ‘information power’ ([Sample Size in Qualitative Interview Studies: Guided by Information Power - Kirsti Malterud, Volkert Dirk Siersma, Ann Dorrit Guassora, 2016 (sagepub.com)](https://journals.sagepub.com/doi/abs/10.1177/1049732315617444?journalCode=qhra)). Moreover, we applied Francis and colleagues’ (2010) recommendations to determine sample size for a theory-informed qualitative descriptive study (<https://pubmed.ncbi.nlm.nih.gov/20204937/>). Also, as a background reading, we reviewed other widely discussed ideas and recommendation around the concept of data saturation in qualitative research such as the Braun and Clerck’s argument ([To saturate or not to saturate? Questioning data saturation as a useful concept for thematic analysis and sample-size rationales: Qualitative Research in Sport, Exercise and Health: Vol 13, No 2 (tandfonline.com)](https://www.tandfonline.com/doi/abs/10.1080/2159676X.2019.1704846).  Unlike quantitative designs, the qualitative approaches do not have straightforward rules of sample size calculation. Instead, they depend on data adequacy guided by certain principles such as ‘saturation’ and ‘information power’. In our case, as we used purposive sampling to select ‘information rich’ participants (healthcare providers who told their experiences in a well articulated manner, we collected rich or adequate data by interviewing 13 participants. A recent systematic review that assessed saturation in qualitative studies shows that 9-17 interviews reached saturation ([Sample sizes for saturation in qualitative research: A systematic review of empirical tests - ScienceDirect](https://www.sciencedirect.com/science/article/pii/S0277953621008558)).  On the other hand, this qualitative descriptive study focuses on a comprehensive understanding of healthcare providers' perceptions and behaviors without attempting to identify consistency or differences among each group of healthcare providers. Instead, it identifies commonalities and enriches data from different perspectives and contexts. Therefore, sub-group analysis was not our aim.  Regarding the recommendation on the application of standard tools and methods, we are confident that we applied all the requirements for a qualitative descriptive study and applied the Consolidated criteria for reporting qualitative studies (COREQ) ([Consolidated criteria for reporting qualitative research (COREQ): a 32-item checklist for interviews and focus groups \| EQUATOR Network (equator-network.org)](https://www.equator-network.org/reporting-guidelines/coreq/)). Unlike quantitative studies, qualitative studies do not utilize standardized tools or instruments for data collection. Therefore, we did not use a standardized tool; instead, we used an interview guide developed by the research team and refined after pilot interviews. We used a published method article for data analysis and followed all the steps recommended. Our methodology and methods are explicitly described on pages 5-10 of the manuscript.  One of the four questions presented to the reviewers does not apply to our study design. For the question “Has the statistical analysis been performed appropriately and rigorously?” reviewer #1 said “No,” whereas reviewer #2 said “Yes.” However, statistical analysis is not applicable in our qualitative study. |
