# Supplementary material for: Perceptions and behaviors of healthcare providers towards rehabilitation support to children with severe malaria-related disability in Ethiopia: A qualitative descriptive study using the Theoretical Domains Framework
Source: PLoS One. 2024 May 2;19(5):e0298769. doi: 10.1371/journal.pone.0298769 (PMC11065226; doi:10.1371/journal.pone.0298769)
Supplement: Attachment — Submitted filename: Response to Reviewers.docx [file pone.0298769.s008.docx]

**Response to Reviewer #2**

| **Reviewer’s comment/question** | **Authors’ response** |
| --- | --- |
| The authors have responded adequately to all my questions, and I am satisfied with this revision. | Thank you so much for your time and scholarly feedback; your comments helped us improve our manuscript. |
| Regarding their comment on the question that asks whether statistical analysis has been performed appropriately and rigorously where reviewer 1 responded “No” and reviewer 2 “Yes” As the 2nd reviewer, my initial response “Yes” to this was based on interpretation of this question in the context of a qualitative study to be asking whether the qualitative analysis had been performed appropriately to acceptable standards for reproducibility of this research work. | Yes, that makes sense. |

**Responses to Reviewer #3**

| **Reviewer’s comment/question** | **Authors’ response** |
| --- | --- |
| One minor suggestion in line 163: non-verbal expressions of participants, and his feelings (50). I suggest that you delete "his" | Thank you so much for your time and comment. The pronoun ‘his’ has been deleted. |

**Responses to Reviewer #4**

| **Reviewer’s comment/question** | **Authors’ response** |
| --- | --- |
| Thank you for submitting your well-written manuscript for review. I appreciate your careful research and the insights you've provided. However, I have some suggestions, questions, and corrections that I believe will further strengthen your manuscript. | Thank you so much for your rigorous review and detailed comments. |
| Abbreviations: Please check the consistency of abbreviations throughout the manuscript, especially at L54. Ensure that all abbreviations are defined clearly at their first mention. | We have re-checked the abbreviations throughout the document. We used abbreviations when a text that needs to be abbreviated appears more than three times. In line 54, the World Health Organization appears only twice, and we prefer to spell it out instead of using the abbreviation WHO. |
| ICF Abbreviation: On page 62, please use the correct abbreviation. | The standard abbreviation for the ‘International Classification of Functioning Disability and Health’ is ICF, as abbreviated by the World Health Organization (WHO). Please see in the following link: [International Classification of Functioning, Disability and Health (ICF) (who.int)](https://www.who.int/standards/classifications/international-classification-of-functioning-disability-and-health) |
| Citations: The current citation style needs to be refined to adhere to the journal's guidelines. Please review the citation style guide and ensure that all references are cited correctly. | We used the Vancouver referencing style as highlighted in PLOS ONE’s authors’ guideline: “PLOS uses the reference style outlined by the International Committee of Medical Journal Editors (ICMJE), also referred to as the “Vancouver” style.” As we also used the EndNote citation manages, we believe that the references have been inserted correctly. |
| Contributions: While I understand that the EE played a significant role in the practical aspects of the study, I recommend adding a contribution statement at the end of the manuscript. This will provide a clear overview of each author's contribution to the research. | A contribution statement has been added at the end of the manuscript. Please see the tracked changes (L776). |
| Reference Clarification: At L163, please provide more specific information about the reference you've cited. | The following clarification is added ‘as recommended by Phillipi and Lauderdale (2018).’ |
| Verbatim Software: At L164, please include the company name and country of origin for the Verbatim software mentioned. | We did not use software for the verbatim transcription; instead, research assistants transcribed it. This clarification is added to the document. |
| NVivo Software: At L167, please add more context about the NVivo software, such as its purpose and applications. | We re-phrased the section through L167-169 to address your comment: “We imported the transcripts into NVivo 12 Plus software to facilitate the data analysis. The purpose of the software was to assist the researchers in organizing, visualizing, storing, and reporting their data; otherwise, the entire analysis was a cognitive process.” |
| Amharic: Please explicitly mention the language after the first time Amharic is used in the manuscript, ensuring clarity for readers. | We added the statement described below (with references) to clarify how we used Amharic (the local language).  “To minimize loss of meaning during translation, we analyzed the data using Amharic (the local language) until we generated preliminary themes. After that, the codes and associated data were translated into English.” (L170-172) |
| Health Care Selection: I'm curious about the basis for selecting the health care facilities you've included in your study. Please provide more details about your selection criteria. | In line with the nature of the qualitative design, we selected both the setting and participants purposively with criteria. The criteria for selecting the healthcare facilities were (1) their level (primary, secondary, and tertiary), and (2) healthcare facilities found in malaria endemic areas. These criteria are included in the revised version (L138-139). |
| Health Care Availability: Can you provide information about the total number of health care facilities in the country and explain why you chose to focus on these specific ones? Are they representative of the overall health care landscape? | Unlike quantitative studies, which commonly aim for generalization, the qualitative approach does not aim for that, as generalizing the findings is not its purpose. Instead, it aims for transferability, defined as “The degree to which the results of qualitative research can be transferred to other contexts or settings with other respondents. The researcher facilitates the transferability judgment by a potential user through thick description.” ([Series: Practical guidance to qualitative research. Part 4: Trustworthiness and publishing (tandfonline.com)](https://www.tandfonline.com/doi/pdf/10.1080/13814788.2017.1375092); p. 121). Collecting ‘representative’ samples is associated with generalizability and sounds like a post-positivist axiology, which differs from our naturalistic perspective in which collecting a representative sample is less valued. Instead, we choose samples purposively and keep recruiting participants until data and meaning saturation happens and adequacy of information is ensured.  Therefore, in line with our philosophical worldview, we did not consider representativeness and generalizability but transferability. What is critical to ensure transferability, in addition to the behaviors of the healthcare providers, is the context related to the phenomenon under investigation. Therefore, we added a detailed description of the context of the Ethiopian healthcare facilities in relation to malaria treatment and care. Please see L140 of the revised version in the tracked changes. |
| I believe that addressing these suggestions, questions, and corrections will further enhance the manuscript's clarity, rigor, and overall quality. Thank you for your attention to these details. | Yes, your comments are indispensable and help us improve our manuscript. Thank you so much. |

**Responses to Reviewer #5**

| **Reviewer’s comment/question** | **Authors’ response** |
| --- | --- |
| Minor grammatical and clarification of some sentences needed. Further comments are included in the article which has been uploaded. | Thank you so much for your comments. We have corrected the grammatical issues and clarified some sentences in the document per your suggestions. The changes we made are described below and in tracked changes in the manuscript. |
| L230 Table 2: General Practitioners; qualify if they are Physician General Practitioners | Yes, they are Physician General Practitioners, and it is corrected in the document. |
| L238 “We also delineated the proportion of codes addressed the prevention of severe malaria-related disability” Clarify this | Clarification added, and the modified statement is as follows: “We also delineated the proportion of codes that addressed the prevention of severe malaria-related disability, indicating acute care of severe malaria instead of rehabilitation support and care.” |
| L396 “On the other side of the spectrum, many participants described that the focus of the health facilities could be diverted when new public health important disease emerge, and this factors negatively influence the amount of budget allocated for malaria-related activities.” re-phrase; grammatical error | It is corrected, and the sentence has been re-phrased as follows: “On the other side of the spectrum, many participants described that the focus of the health facilities could be diverted when new pandemics or epidemics emerge, and these factors negatively influence the budget allocated for malaria-related activities.” |
| L506-507 “Our findings show that the healthcare providers 24 507 have not been systematically provided rehabilitation support for children with severe malaria.” Clarify this sentence. | The sentence is re-phrased for clarification as follows:  “Our findings show that the healthcare providers have not been provided rehabilitation support for children with severe malaria as part of the national malaria treatment and care protocol.” |
| L516-518 “The main gap in the referral system was a lack of feedback about when and on what condition severe malaria survived children were returned to their home or to their community, which created an information gap regarding the frequency and type of health check-ups.” In the phrase “when and on what condition” in or on? | It should be ‘in.’ Corrected in the document. |
| L533 “What worsens the problem is the socio-cultural influences circumscribing some severity signs such as seizures, delirium, and coma.” How do the sociocultural issues restrict some severity signs? | It is explained in the next sentence in the document (L533-534), highlighting that “Parents in rural areas, in general, and some people in urban areas associate these issues with “evil eyes” or “evil spirits” and do not take children with those symptoms to a modern health facility.” |
| L540 “On the contrary …” to what? | The phrase “On the contrary” is replaced by “On the other hand.” |
